# NetLnc: A Network-Based Computational Framework to Identify Immune Checkpoint-Related lncRNAs for Immunotherapy Response in Melanoma

**DOI:** 10.3390/ijms26104557

**Published:** 2025-05-09

**Authors:** Qianyi Lu, Jian Li, Wenli Chen, Zhuoru Wang, Di Wang, Chenyu Liu, Yue Sun, Han Jiang, Caiyu Zhang, Yetong Chang, Jiajun Zhou, Xiaohong Wu, Yue Gao, Shangwei Ning

**Affiliations:** 1 College of Bioinformatics Science and Technology, Harbin Medical University, Harbin 150081, China; 2 NHC and CAMS Key Laboratory of Molecular Probe and Targeted Theranostics, Harbin Medical University, Harbin 150081, China; wuxiaohong@hrbmu.edu.cn

**Keywords:** immune checkpoint, long non-coding RNA, immunotherapy, melanoma, biomarker

## Abstract

Long non-coding RNAs (lncRNAs) could alter the tumor immune microenvironment and regulate the expression of immune checkpoints (ICPs) by regulating target genes in tumors. However, only a few lncRNAs have precise functions in immunity and potential for predicting ICP inhibitors (ICI) response. Here, we developed a computational multi-step framework that leverages interaction network-based analysis to identify cancer- and immune-context ICP-related lncRNAs (NetLnc). Based on bulk and single-cell RNA sequencing data, these lncRNAs were significantly correlated with immune cell infiltration and immune expression signature. Specific hub ICP-related lncRNAs such as *BANCR*, *MIAT*, and *SNHG15* could predict three- and five-year prognosis of melanoma in independent datasets. We also validated that some NetLnc-based predictions could better effectively predict ICI response compared to single molecules using three kinds of machine learning algorithms following independent datasets. Taken together, this study presents the use of a network-based framework to efficiently select ICP-related lncRNAs, which contributes to a comprehensive understanding of lncRNA functions and accelerates the discovery of lncRNA-based biomarkers in ICI treatment.

## 1. Introduction

The use of immune checkpoint inhibitors (ICIs) is an important method in tumor immunotherapy which can block the binding of immune checkpoint (ICP) receptors to their ligands, thus blocking the inhibition by tumor cells of immune cells and activating immune cells to play an anti-tumor role [1]. Melanoma is a highly aggressive malignant tumor with limited efficacy of conventional treatments, and the use of immune checkpoint inhibitors has significantly improved patient survival rates [2]. Many ICI treatments for patients with advanced melanoma or those who have developed distant metastases have significantly improved patient prognosis [3]. However, in some patients, drug resistance and immune-related adverse events occur, which can be fatal in a fraction of patients [4]. Therefore, it is urgent to identify accurate biomarkers for predicting response to ICI.

Long noncoding RNAs (lncRNAs) are a class of non-coding transcripts with more than 200 nucleotides involved in the regulation of cell differentiation and development [5]. They are involved in the regulation of chromatin state and gene expression through various mechanisms, including epigenetic, transcriptional, and post-transcriptional regulation [6]. LncRNAs play complex roles in the tumor microenvironment, participating in tumor development [7], promoting malignant tumor progression [8], and also enhancing the tumor immune response. In tumor immune response, lncRNAs can regulate the function of immune cells and affect the immune escape from tumors and the efficacy of immunotherapy [9,10,11]. Expression levels of lncRNAs are often abnormal, proving them to be potential biomarkers for early cancer diagnosis and potential targets for cancer therapy [9]. A growing number of studies have shown that lncRNAs play a role in predicting and evaluating the response to immune checkpoint inhibitor therapies as possible biomarkers [12]. However, a comprehensive assessment of the mechanisms by which most lncRNAs predict ICI treatment is still lacking.

The development of next-generation sequencing has allowed us to characterize tumor heterogeneity at the gene level, and some previous studies have constructed immunotherapy prognostic models. For example, Da Liu et al. constructed a prognostic assessment tool based on cuproptosis-related genes that could predict the prognosis and drug sensitivity of cutaneous melanoma [13]. Can Lu et al. constructed a prognostic model by characterizing the molecular characteristics of microsatellite stable colorectal cancer, which could predict prognosis and immunotherapeutic response [14]. More and more evidence has reported that lncRNAs can directly or indirectly regulate ICP molecules to affect ICI therapy. For example, Qingyu Lin et al. found that *HITT* plays a role in anti-tumor immunity by inhibiting PD-L1 translation through binding to the regulatory protein RGS2 [15]. However, these studies rarely constructed models to predict prognosis and immunotherapy from the perspective of ICP-related lncRNAs.

In this study, we report an immune-context computational multi-step framework that leverages network-based analysis inference to (i) identify ICP-related lncRNAs and (ii) identify potential biomarkers. We summarized the patterns of immune-related lncRNAs regulation in melanoma. The identified results were validated in independent datasets and databases. We found that common and specific ICP-related lncRNAs were involved in diverse immune processes. ICP-related lncRNAs were correlated with immune cell infiltration in melanoma patients based on bulk and single-cell RNA sequencing. Using our network-based ICP-related lncRNAs improved the prediction of the overall survival in melanoma patients. We found that the NetLnc-based predictions were more accurate than predictions based on the expression levels of ICI targets including PD1, PD-L1, or CTLA4 based on multiple independent datasets. In conclusion, our computational framework provides an approach to unveil ICP-related lncRNAs, opening new avenues to investigate biomarkers for ICI therapy in melanoma patients and helping previously identified biomarkers improve the prediction of ICI response.

## 2. Results

### 2.1. Overview of a Computational Framework for Identifying ICP-Related lncRNAs

**Figure 1:**
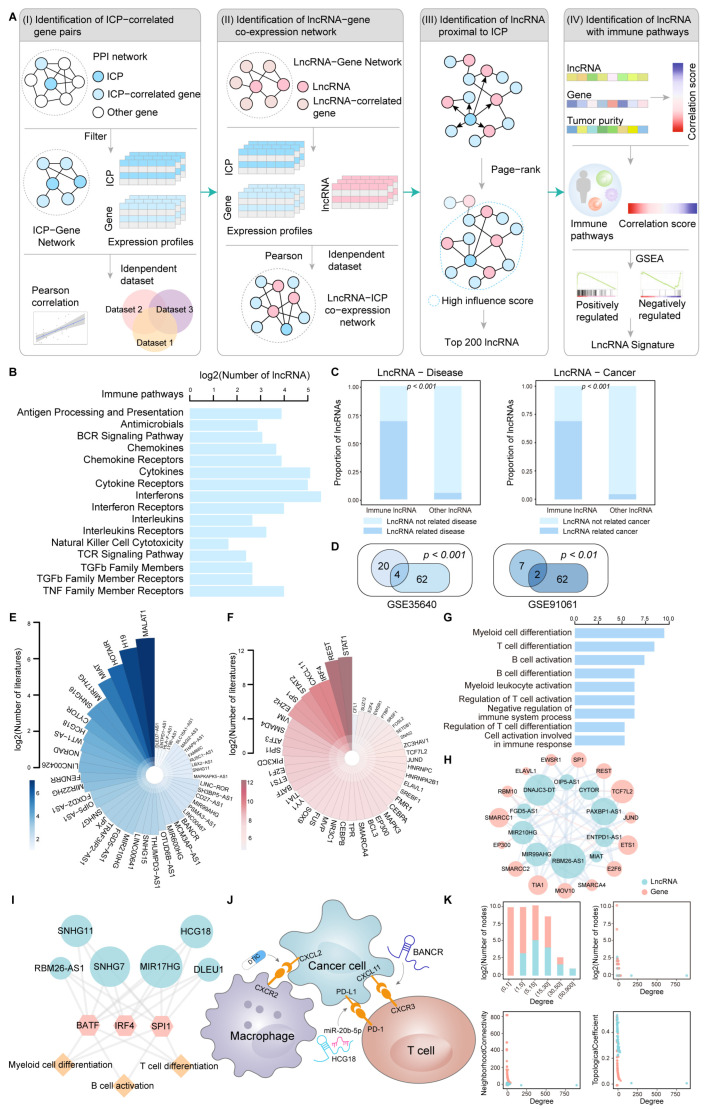
Overview of a computational framework for identifying ICP-related lncRNAs. (**A**) Schematic illustration of four steps for the identification of ICP-related lncRNAs. (**B**) The number of lncRNAs in immune-related pathways. (**C**) The validation of immune-related lncRNAs in various databases. The figure on the left shows the validation of immune-related lncRNAs in the LncRNADisease database. The figure on the right shows the validation of immune-related lncRNAs in the Lnc2Cancer database. The chi-squared test was used to evaluate the difference. (**D**) Venn diagrams show the results identified with other independent datasets. The intersections represent jointly identified results. The hypergeometric test was used to assess the significance of the overlap. (**E**) A Nightingale rose chart shows the number of lncRNAs that co-occurred with ‘immune’ in the literature. (**F**) A Nightingale rose chart shows the number of genes that co-occurred with ‘immune’ in the literature. (**G**) The bar plot shows the pathways involved in immune-related genes. (**H**) An example of a sub-network of lncRNAs and genes in a co-expression network. (**I**) An example of a sub-network of lncRNAs, genes, and regulated immune functions in melanoma. Green, red, and blue indicate lncRNA, ICP genes, and immune functions, respectively. (**J**) A diagram shows the interactions between immune cells and tumor cells modulated by two ICP-related lncRNAs. The target genes regulated by ICP-related lncRNAs shown in the plot are receptors and ligands expressed on the surface of immune cells or tumor cells. (**K**) Network topological properties of the lncRNA-ICP gene co-expression network.

To identify lncRNAs associated with immune checkpoints, we proposed a four-step computational framework (Figure 1A). The framework systematically infers ICP-related lncRNAs and their regulatory mechanisms from expression profiles, network modules, and immune-related pathways based on a large number of samples. Briefly, four steps were used to identify ICP-related lncRNAs. First, we collected human protein–protein interaction network and ICP. Second, based on the previously collected lncRNA–gene interaction network, we obtained lncRNAs correlated with ICP and ICP genes and screened genes with strong mutual correlations through Pearson correlation analysis. Third, we used the page-rank algorithm to identify lncRNAs closely associated with ICP. Finally, we further identified lncRNAs enriched in immune-related pathways based on lncRNA expression, gene expression, and tumor purity for each patient.

We focused on 17 immunologically relevant gene sets representing distinct immune pathways from ImmPort. A total of 62 lncRNAs were involved in these pathways (Figure 1B). We found that these immune-related lncRNAs are likely to co-occur with disease and cancer in the databases (Figure 1C). These results imply that they might play key roles in tumor-related pathways. Similar results were validated in other independent datasets (Figure 1D). We found that ICP-related lncRNAs and genes were generally recognized as being immunologically relevant in the Pubmed database (Figure 1E,F). To further explore the functional characteristics of lncRNAs and genes, enrichment analysis was performed. We found that a number of functions are related to immunity, including myeloid cell differentiation, T-cell differentiation, and B-cell activation (Figure 1G,H). We surprisingly found that ICP-related lncRNAs could regulate genes mediating the interactions between tumor cells and immune cells, such as T cells (Figure 1J). *BANCR* knockdown reduces melanoma cell migration by upregulating the chemokine CXCL11 [16]. Treatment with the anti-tumor drug Decarbazine (DTIC) reduced melanoma cell migration by decreasing protein concentrations of the migrating chemokines CXCL2 and CXCL11 [17]. Mechanistically, *HCG18* increased PD-L1 expression by sponging *miR-20b-5p* [18]. Moreover, we found that lncRNAs with more neighbors in the network tended to regulate more immune-related functions (Figure 1K). Taken together, our findings may provide a valuable resource in terms of the tumor immune microenvironment.

### 2.2. ICP-Related lncRNAs Are Correlated with Immune Cell Infiltration

**Figure 2:**
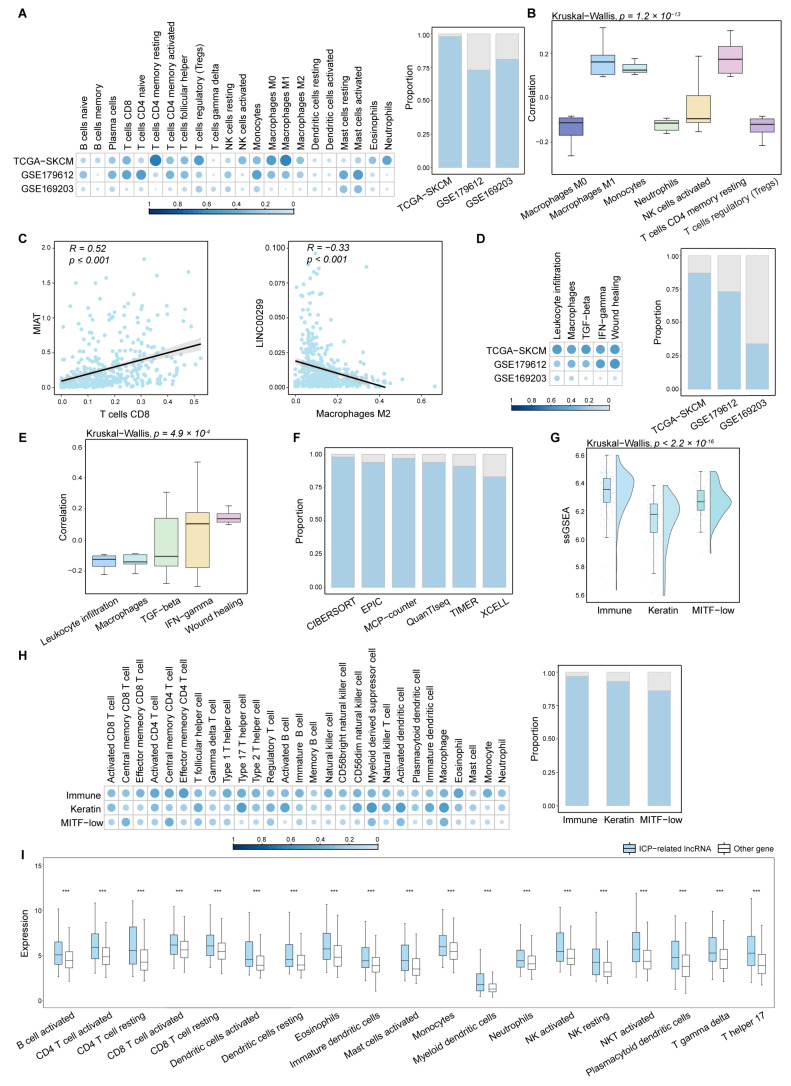
ICP-related lncRNAs are correlated with immune cell infiltration. (**A**) The heatmap shows the proportion of lncRNAs of which expression are significantly related with immune cell infiltration in each dataset calculated by CIBERSORT. The bar plot shows the number of lncRNAs whose expression is significantly related to immune cell infiltration as a proportion of the total number of all lncRNAs in each independent dataset. (**B**) The Spearman’s rank correlation coefficients between the expression of lncRNAs and immune cell infiltration. The box plots are shown as median (line), interquartile range (box), and data range. The Kruskal–Wallis test was used. (**C**) Examples of two lncRNAs closely related to immune cell infiltration. The fitting curve was performed with the lm function. Each blue dot represents correlation coefficient between ICP-related lncRNA expression and immune cell infiltration. The black line represents the fitting curve. The gray area represents confidence intervals. (**D**) The heatmap shows the proportion of lncRNAs whose expression is significantly related to immune expression signatures in each independent dataset. The bar plot shows the number of lncRNAs whose expression is significantly related to immune expression signatures as a proportion of the total number of all lncRNAs in each dataset. (**E**) The Spearman’s rank correlation coefficients between the expression of lncRNAs and immune expression signatures. The box plots are shown as median (line), interquartile range (box), and data range. The Kruskal–Wallis test was used. (**F**) The bar plot shows the number of lncRNAs whose expression is significantly correlated with immune cell infiltration as a proportion of the total number of all lncRNAs. The level of immune cell infiltration was calculated by different algorithms. (**G**) The raincloud plot shows ssGSEA scores with lncRNAs and ICPs in ‘immune’, ‘keratin’, and ‘MITF-low’ clusters of SKCM. The Kruskal–Wallis test was used. (**H**) The heatmap shows the proportion of lncRNAs whose expression is significantly related to immune cell infiltration in each subtype calculated by ssGSEA. The bar plot shows the number of whose expression is significantly related to immune cell infiltration as a proportion of the total number of all lncRNAs in each subtype. (**I**) Expression of ICP-related lncRNAs and other genes in immune cell lines. The Wilcoxon Mann–Whitney test was used. *** *p* < 0.001.

We reasoned that if ICP-related lncRNAs function in the tumor microenvironment, it is possible that they are highly expressed in immune cells and correlate with immune cell infiltration in tumors. There is a strong correlation between lncRNAs and ICPs (Supplementary Figure S1). Immune cell infiltration levels were estimated by CIBERSORT based on lncRNA expression profiles, and then we analyzed the associations between the expression of lncRNAs and immune cell infiltration levels. We found that a number of ICP-related lncRNAs were correlated with immune cell infiltration (Figure 2A). In particular, this correlation was higher in macrophages, monocytes, and T cells (Figure 2B). For example, the correlation coefficient between *MIAT* expression and CD8 T cells was as high as 0.55 (Figure 2C). *LINC00299* were correlated with macrophages M2 cells infiltration with correlation coefficients −0.33. We then calculated the associations of lncRNA expression with five immune expression signatures. We similarly found that many ICP-related lncRNAs were correlated with immune expression signatures (Figure 2D), especially IFN-γ response and wound healing (Figure 2E). We further used a variety of algorithms for calculating the level of immune cell infiltration, all of which yielded a large number of ICP-related lncRNAs significantly correlated with immune cell infiltration (Figure 2F).

SKCM patients in TCGA could be clustered to three subtypes including ‘immune’, ‘keratin’, and ‘MITF-low’ clusters [19]. A significant number of genes in the immune cluster were associated with immune cell subsets and immune signaling molecules. In our analysis, the immune cluster is most strongly associated with lncRNA and ICP (Figure 2G) and is most significantly correlated with immune cell infiltration (Figure 2H). Then, we analyzed the differences in expression of ICP-related lncRNAs versus other genes in 19 immune cell lines. We found that the expression of ICP-related lncRNAs was generally higher (Figure 2I). Collectively, these results suggest that ICP-related lncRNAs exhibit higher expression in immune cells and are correlated with immune cell infiltration, further validating the roles of the ICP-related lncRNAs in the tumor-immune microenvironment.

### 2.3. ICP-Related lncRNAs Showed Specific Features Across Immune Cell Subsets in scRNA-Seq Data

**Figure 3:**
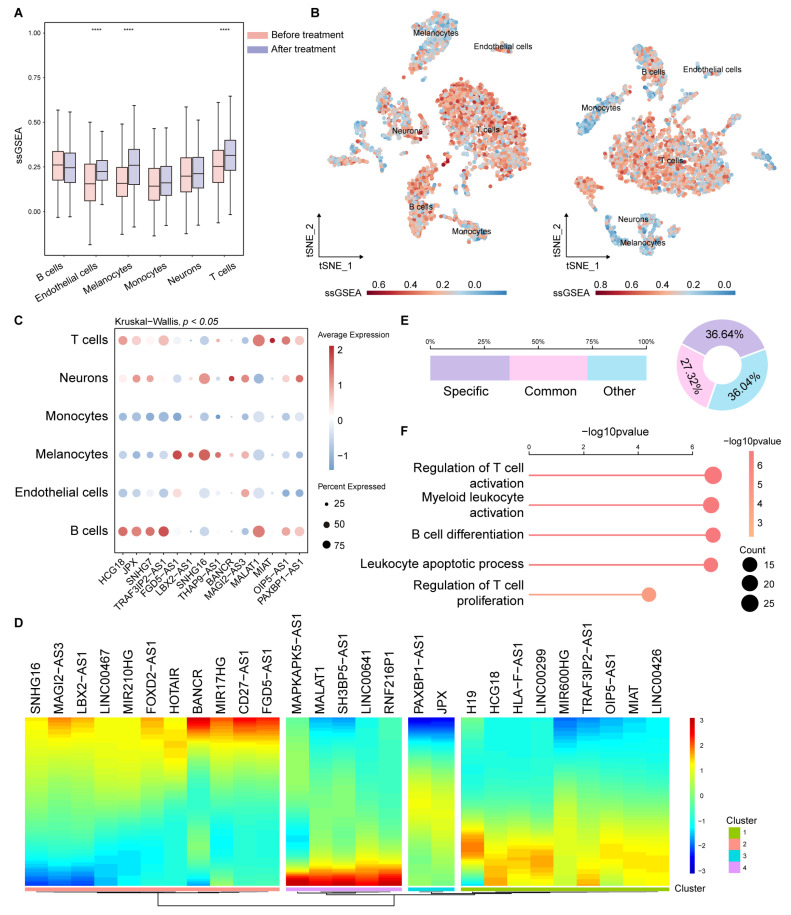
ICP-related lncRNAs showed specific features across immune cell subsets based on single-cell RNA sequencing. (**A**) ssGSEA scores of lncRNAs in melanoma before and after immunotherapy. The box plots are shown as median (line), interquartile range (box), and data range. The Wilcoxon Mann–Whitney test was used. **** *p* < 0.0001. (**B**) tSNE plots of cells in melanoma before and after immunotherapy, color-coded by ssGSEA scores for lncRNAs. (**C**) The dot plot shows ICP-related lncRNAs expression in different cell types. The Kruskal–Wallis test was used. (**D**) The heatmap shows pseudotime of ICP-related lncRNAs in melanoma. (**E**) The bar plot and chart show the percentage of lncRNA-gene pairs with different patterns. (**F**) The plot shows the pathways involved in specific pattern pairs. **** *p* < 0.0001.

We further analyzed the scRNA-seq data to explore the roles of ICP-related lncRNAs in the tumor immune microenvironment and ICI treatment regulation. We collected patients with pre-treatment and post-treatment immunotherapy data for comparison. The major cell types included B cells, endothelial cells, melanocytes, monocytes, neurons, and T cells (Supplementary Figure S2A). We found that ssGSEA scores of lncRNAs were different in endothelial cells, melanocytes, and T cells (Figure 3A,B). In addition, ICP-related lncRNAs were differentially expressed in different cell types (Figure 3C). These ICP-related lncRNAs were dynamically changed in diverse pseudotime points (Figure 3D). All cells formed a branched structure with two transcriptional states based on expression profile (Supplementary Figure S2B).

In addition, we estimated the correlation between lncRNAs and ICP in different cell types. Based on the correlation patterns presented by lncRNA-ICP pairs in different cell types, we binned these pairs in three categories: specific (pairs with correlation in only one cell type, accounting for 36.64%), common (pairs with correlation in multiple cell types, accounting for 27.32%), and other (the rest of pairs, accounting for 36.04%) (Figure 3E). We found that lncRNA–gene pairs presenting specific patterns had the highest ssGSEA scores, which may indicate that they have a greater impact on the immune microenvironment (Supplementary Figure S3). Thereby, we found these specific pattern pairs are related with immune function, including regulation of T cell activation, myeloid leukocyte activation, and B-cell differentiation (Figure 3F). In summary, these results suggested that ICP-related lncRNAs are associated with immune cell infiltration, further validating the roles of the ICP-related lncRNAs in the tumor-immune microenvironment.

### 2.4. Several ICP-Related lncRNAs Are Associated with Survival in Melanoma Patients

**Figure 4:**
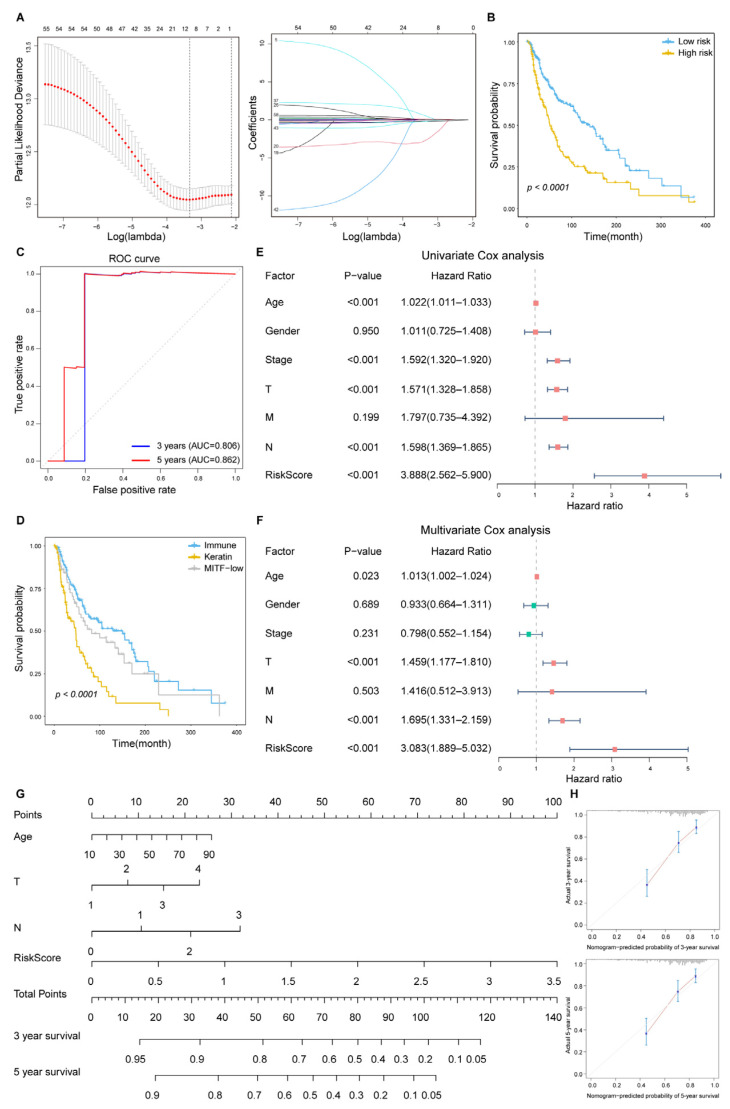
Several ICP-related lncRNAs are associated with survival in melanoma patients. (**A**) Left, ten-time cross-validation for tuning parameter selection in the LASSO model. Right, LASSO coefficient profiles. (**B**) Kaplan–Meier survival analysis of OS for patients with high (yellow) and low (blue) risk scores. The survival difference is calculated by the log-rank test. (**C**) ROC curves for 3-year and 5-year OS prediction of ICP-related lncRNAs were performed in TCGA SKCM. (**D**) Kaplan–Meier survival analysis of OS in ‘immune’ (blue), ‘keratin’ (yellow), and ‘MITF-low’ (grey) clusters of SKCM. (**E**,**F**) Forest plots of univariate and multiple Cox regression analyses. Each horizontal line indicates a 95% confidence interval. The dashed line is the null line, indicating OR = 1. Squares indicate point estimates. Red squares represent OR > 1 and green squares represent OR < 1. (**G**) Nomogram to estimate the prognostic risk of ICP-related lncRNAs. Each variable axis corresponds to the characteristic attribute score of a single sample. The likelihood of 3-year and 5-year OS is determined on the survival axis. (**H**) The calibration curves show the predictive capability of ICP-related lncRNAs. The *X*-axis represents the predicted value of survival probability and the *y*-axis represent actual survival possibility.

We next investigated whether these ICP-related lncRNAs were associated with the survival of cancer patients. According to our analysis, some ICP-related lncRNAs could serve as prognostic biomarkers in melanoma (Figure 4A). A low risk score was associated with a better prognosis in SKCM patients (*p* = 4.85 × 10^−5^; Figure 4B). ROC curves were employed to assess the accuracy of established models for predicting OS in patients. The AUC values for 3 years and 5 years were 0.806 and 0.862, respectively, indicating the robustness and accuracy of risk score in predicting patient prognosis (Figure 4C). Melanoma patients had a better prognosis in the immune cluster (Figure 4D). We further performed univariate and multivariate Cox regression analysis. Univariate Cox regression analysis showed that risk score was a prognostic predictor in SKCM (Figure 4E). More importantly, the risk score was also observed to be an independent predictor in multivariate Cox regression analysis (Figure 4F). Based on the independent predictors obtained from the multivariate Cox analysis, a nomogram was established to predict the 3-year and 5-year survival (Figure 4G). The calibration curve showed that risk score had a satisfactory fit between the predicted and actual observations (Figure 4H). Analysis of multiple independent datasets suggests that risk scores could generally predict prognosis in melanoma patients (Supplementary Figure S4). Thus, the risk score could effectively predict the 3-year and 5-year prognosis in SKCM patients. Taken together, these results indicated that risk score based on the expression of ICP-related lncRNAs could effectively identify prognostic biomarkers for cancers.

### 2.5. A Few ICP-Related lncRNAs Could Improve the Prediction of Immunotherapy Response in Melanoma Patients

**Figure 5:**
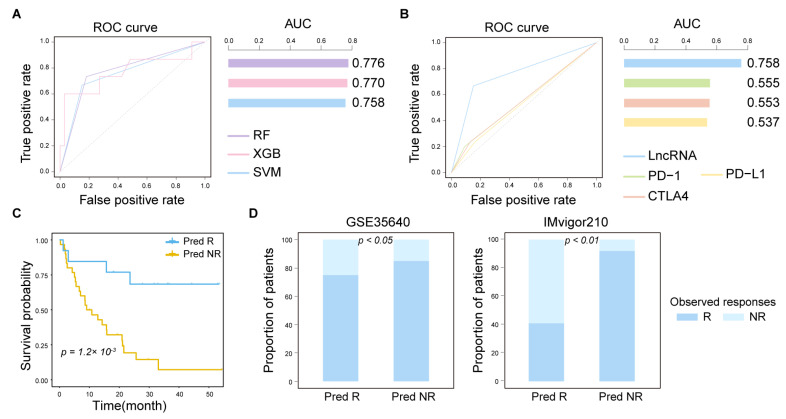
A few ICP-related lncRNAs could improve the prediction of immunotherapy response in melanoma patients. (**A**) ROC curves of three machine learning algorithms constructed using ICP-related lncRNAs to predict ICI response. (**B**) Comparison of the ability of ICP-related lncRNAs to predict ICI response with that of other clinical biomarkers. (**C**) Kaplan–Meier survival analysis of OS in melanoma patients for responders (blue) and non-responders (yellow). The survival difference is calculated by the log-rank test. (**D**) Immunotherapy-response prediction using the expression levels of ICP-related lncRNAs. Predicted responders (Pred R) and non-responders (Pred NR) are plotted against observed responders (blue) and non-responders (light blue). The two-sided Fisher’s exact test was used to compute statistical significance.

To evaluate the predictive performance of ICP-related lncRNAs for ICI response, we integrated multiple datasets from melanoma for analysis. We constructed three kind of machine learning algorithms, including random forest, extreme gradient boosting, and support vector machine. We found that the ROC values were all greater than 0.75, especially for random forest (AUC = 0.776; Figure 5A). We also compared the ability of ICP-related lncRNAs with clinical biomarkers in predicting response. The AUC values of ICP-related lncRNAs were all greater than those of clinical biomarkers, including PD-1, PD-L1, and CTLA4 (AUC = 0.758; Figure 5B). Furthermore, a prolonged overall survival was consistently observed for patients predicted as ICI responders using ICP-related lncRNAs (Figure 5C). ICP-related lncRNAs were established in immunotherapy datasets to evaluate their predictive value (Figure 5D). ICP-related lncRNAs can also be predictive of prognosis in other diseases. Collectively, our results suggest that ICP-related lncRNAs enable robust prediction of immunotherapy.

## 3. Discussion

Dysfunction of ICPs in tumor cells enables them to evade tumor immunosurveillance and to suppress anti-tumor immune responses, promoting tumor development and progression [20]. Increasing evidence suggests that lncRNAs can directly or indirectly regulate ICP genes. Certain lncRNAs modulate the efficacy of immunotherapy by altering the infiltration patterns of immune cells in the tumor microenvironment through interactions with immune checkpoint molecules. A previous study by Qing Xi et al. demonstrated that *TUG1* promotes immune escape in hepatocellular carcinoma by regulating the expression of PD-L1 and CD47 while inhibiting the activation of CD8+ T cells and macrophage phagocytosis [21]. Therefore, systematic analysis of the potential of ICP-related lncRNAs in predicting prognosis and ICI response is crucial for exploring the regulatory mechanisms of ICP molecules in tumors. Herein, a multi-step computational framework was developed to identify ICP-related lncRNAs in melanoma (Figure 1A). We investigated ICP-related lncRNAs to understand the role of lncRNAs in ICI treatment. Our results suggest that ICP-related lncRNAs could predict patient prognosis and immunotherapy response. There is a complex relationship between ICP, lncRNAs, and immunity, and this may be helpful in evaluating ICI response in cancer patients.

To further understand the role of lncRNAs in melanoma immunoregulation, we identified lncRNAs correlated with ICP and ICP genes. The page-rank algorithm was used to extract ICP-proximal lncRNAs. The top 200 lncRNAs with high scores were identified in our work. Next, we proposed to identify lncRNAs enriched in immune-related pathways for further screening. In our analysis, we focused on 17 immunologically relevant gene sets representing distinct immune pathways from ImmPort. Many melanoma patients have seen significant clinical outcomes with immunotherapeutic interventions. However, some patients have different responses when provided the same treatment. Previous studies have demonstrated that ICP genes play crucial roles not only in tumor cells but also in tumor-infiltrating immune cells. TIM-3 inhibits T-cell activity and proliferation, promotes immune tolerance, and reduces the anti-tumor effect of T cells by binding to its ligands, such as Galectin-9 [22]. CD47 binds to the SIRPα receptor on macrophages, inhibiting macrophage phagocytosis and preventing the immune system from clearing tumor cells. Tumor cells exploit this pathway to evade recognition and phagocytosis by macrophages by upregulating CD47 expression [23]. These ICP genes significantly influence the immune microenvironment through interactions between immune cells and tumor cells. Variations in the responsiveness of different immune cell subpopulations to ICP may contribute to the differences in immunotherapy response observed in melanoma patients. The diversity of immune regulation in tumors may affect the efficacy of immunotherapy, and ICP-related lncRNAs based on ICP may provide a basis for future clinical immunotherapy.

Melanoma has been treated with drugs related to immune targets such as CTLA-4, PD-1, and LAG-3. Despite the progress of immunotherapy in melanoma treatment, there are still challenges here, such as the issue of patient resistance or insensitivity to immunotherapy. Although tumor mutational burden, PD-L1, and IFN-γ signature have become relatively well-established biomarkers, these biomarkers lack universality and specificity. This suggests that we need to continuously explore more accurate biomarkers of melanoma immunotherapy efficacy for predicting ICI response. According to our analysis, compared with a single gene, ICP-related lncRNAs could better predict three- and five-year OS in melanoma patients. With respect to the integrated melanoma datasets, ICP-related lncRNAs exhibited better predictive ability than did several clinical biomarkers based on many machine learning algorithms. In future work, the ability of some ICP-related lncRNAs to predict ICI response should be validated in other cancer types. The ICP-related lncRNAs were created using a subset of melanoma patient samples from the TCGA and GEO databases. In addition, ICP-related lncRNAs were generated exclusively through bioinformatics research, and further basic investigations are required to confirm the conclusions.

## 4. Materials and Methods

### 4.1. Data Collection

RNA expression profiles and clinical information of melanoma patients including age, gender, stage, survival time, survival status, and response to immune checkpoint blockade were obtained from The Cancer Genome Atlas (TCGA, https://portal.gdc.cancer.gov/, accessed on 1 November 2022). We also downloaded several independent datasets obtained from Gene Expression Omnibus (GEO, https://www.ncbi.nlm.nih.gov/geo/, accessed on 10 November 2022) to validate the algorithm. Healthy human skin tissue data from Genotype-Tissue Expression (GTEx) were downloaded from the UCSC Xena platform (http://xena.ucsc.edu/, accessed on 11 November 2022). The TCGA datasets were downloaded using the ‘TCGAbiolinks’ R package (Version 2.26.0). The R package ‘sva’ (Version 3.46.0) was used to remove batch effects across multiple independent datasets. Microarray data for 19 immune cell lines were taken from the GEO database with the accession numbers GSE6863 [24], GSE8059 [25], GSE13906 [26], GSE23371 [27], GSE25320 [28], GSE27291 [29], GSE27838 [30], GSE28490 [31], GSE28698 [32], GSE28726 [33], GSE37750 [34], GSE39889 [35], GSE42058 [36], GSE49910 [37], GSE51540 [38], and GSE59237 [39] (Supplementary Table S1).

### 4.2. NetLnc: A Computational Multi-Step Framework to Identify ICP-Related lncRNAs

We developed a computational multi-step framework that leverages interaction network-based analysis to identify cancer- and immune-context ICP-related lncRNAs (NetLnc). The construction of NetLnc comprises four steps:

(i) Identification of ICP-correlated gene pairs

We downloaded the human protein–protein interaction network from the STRING database v.11.5 (https://string-db.org/, accessed on 10 November 2022) [40] and considered links containing ICP. We calculated Pearson correlation (adjusted *p*-value < 0.05) in each independent dataset of melanoma patients.

(ii) Identification of lncRNA-gene co-expression network

Based on our collection of lncRNA-gene interaction network, we obtained lncRNAs correlated with ICP and ICP genes and screened genes with strong mutual correlations through Pearson correlation analysis.

(iii) Identification of lncRNAs proximal to ICPs

We identified lncRNAs that were closely associated with the ICP genes via network propagation using the page-rank algorithm from the NetworkX (Version 3.4.2) Python (Version 3.11.9) module. We used one for ICP genes and zero for all other genes in the network as an input for the personalization parameter in the page-rank algorithm. Pearson correlation coefficients between lncRNAs and genes were used as weights for network edges. Default settings were used for any other parameters for the page-rank algorithm (damping factor = 0.85; maximum iterations = 100; convergence tolerance = 1 × 10^−6^). After network propagation, we considered the top 200 lncRNAs with the highest influence scores as ICP-closely related lncRNAs.

(iv) Identification of lncRNAs with immune pathways

We further identified lncRNAs enriched in immune-related pathways. The expression of lncRNA *i* and gene *j* was defined as L(i) = (l_1_, l_2_, l_3_, …, l_i_, …, l_m_) and G(j) = (g_1_, g_2_, g_3_, …, g_j_, …, g_m_), respectively. The tumor purity scores across m patients were defined as P = (p_1_, p_2_, p_3_, …, p_i_, …, p_m_). We first calculated the partial correlation coefficient (PCC) between the expression of lncRNA *i* and gene *j* by considering the tumor purity as a co-variable:

$$PCC_{\left(ij\right)}=\frac{R_{LG}-R_{LP}*R_{GP}}{\sqrt{1-R_{LP}^{2}}*\sqrt{1-R_{GP}^{2}}}$$

where *R_LG_*, *R_LP_*, and *R_GP_* are the correlation coefficients between the expression of lncRNA *i* and gene *j*, the expression of lncRNA *i* and tumor purity, and the expression of gene *j* and tumor purity, respectively. In addition, we obtained the *p*-value for the PCC, defined as *P(ij)*. For each lncRNA-gene pair, we calculated the rank score (*RS*) as follows:

$$RS_{\left(ij\right)}=-\mathrm{lg}p_{\left(ij\right)}*sign(PCC_{\left(ij\right)})$$

All genes and lncRNAs were ranked based on RS scores and then subjected to enrichment analysis. The enrichment score based on GSEA was defined as ES. *p*-values were adjusted using the False Discovery Rate (FDR). Following a previous study [41], we combined the *p*-value and the ES score to a *lncRES* score:

$$\ln cRES=\left\{\begin{array}{c} 1-2p;\;if\;ES>0\\ 2p-1;\;if\;ES<0 \end{array}\right.$$

Thus, the *lncRES* scores ranged from −1 to 1. We considered lncRNAs with the absolute *lncRES* scores > 0.995 and FDR < 0.05 as significant ones.

### 4.3. Correlations Between ICP-Related lncRNAs and Immune Cell

We obtained gene expression characterization data for 22 immune cell types from the previous study by Newman et al. [42], including seven T cell types, naive and memory B cells, plasma cells, NK cells, and bone marrow subsets. The ‘CIBERSORT’ R package (Version 0.1.0) was used to calculate immune cell infiltration scores (Number of permutations = 1000). We calculated the scores of five immune expression signatures for using single-sample gene set enrichment analysis (ssGSEA). The immune expression signatures (‘Macrophages/monocytes’ [43], ‘Lymphocyte infiltration’ [44], ‘TGF-β response’ [45], ‘IFN-γ response’ [46], and ‘Wound healing’ [47]) were from the previous study by Vesteinn et al. [48]. Spearman rank correlation was calculated between the expression of each lncRNA and immune cell infiltration score and immune expression signature score. Different immune infiltration algorithm scores were from TIMER (http://cistrome.shinyapps.io/timer, accessed on 10 March 2020). The Wilcoxon Mann–Whitney test was used to evaluate the differences between the expression of lncRNAs and other genes in 19 immune cell lines. Kruskal–Wallis test was used to evaluate Spearman’s rank correlation coefficients between the expression of lncRNAs and various immune cell infiltration and immune expression signatures. Comparisons between the three subtypes of melanoma were performed with the Kruskal–Wallis test.

### 4.4. Analysis of scRNA-Seq Data in Melanoma

ScRNA-seq expression profiles of melanoma (GSE115978) [49] was downloaded from GEO. The melanoma patients in GSE115978 were grouped as before- and after-treatment for ICI. The preprocessed gene expression matrix and cell annotation information were encapsulated using the R package ‘Seurat’ (Version 4.3.0.1). Data were filtered to include cells with at least 200 genes and no more than 10% moitochondrial genes. Data were normalized with a scale factor of 10,000 and the ‘LogNormalize’ method. The ‘RunHarmony’ function was used to correct batch effects in individual runs. The ‘FindNeighbors’ function was employed to construct the nearest-neighbor graph, followed by unsupervised clustering using the ‘FindClusters’ function. For visualization, the dimensionality was further reduced using t-distributed Stochastic Neighbor Embedding (tSNE) implemented in the Seurat function ‘RunTSNE’. The ‘GSVA’ R package (Version 1.44.5) was used for ssGSEA to evaluate the gene set enrichment score of each cell. The calculated ssGSEA scores were displayed in the tSNE graph. The difference of ssGSEA scores before and after immunotherapy was calculated by Wilcoxon Mann–Whitney test. The difference in lncRNA expression among different cell types was tested by Kruskal–Wallis test. The ‘monocle’ was used for pseudotime analysis.

### 4.5. Survival Analysis of ICP-Related lncRNAs in Melanoma

We systematically analyzed each significant ICP-related lncRNA to verify whether they were prognostically relevant. Least Absolute Shrinkage and Selection Operator (LASSO) was used to identify candidate prognostic ICP-related lncRNAs by R package ‘glmnet’ (Version 4.1.7). LASSO regression can improve the accuracy and interpretability of the model and can exclude the problem of collinearity between independent variables. We assigned risk scores to each patient based on linear combinations of the expression of each lncRNA weighted by the coefficients. The risk scores for each sample were defined as follows:

$$RiskScore=\sum_{j=1}^{n}\beta_{j}*\exp_{j}$$

where *n* is the number of lncRNAs, *β_j_* is the coefficient from lasso, and *exp_j_* is the expression of lncRNA. Patients were divided into high-risk and low-risk groups based on the median risk score, and their survival was analyzed using the Kaplan–Meier (KM) method. The log-rank test was used to compare the survival curves of two or more groups. The area under the receiver operating characteristic (ROC) curve (AUC) was adopted for analyzing the prognostic predictive value of ICP-related lncRNAs in patients.

Univariate Cox regression analysis was performed to analyze the correlation between risk score and overall survival, and the multivariate Cox analysis was used to evaluate whether risk score could serve as an independent prognostic predictor. In addition, to comprehensively assess patients survival, we constructed a nomogram integrating distinct clinicopathological information. Independent datasets GSE78220 [50] and GSE91061 [51], Van Allen et al.’s cohort [52], and Gide et al.’s cohort [53] were used to validate survival analysis.

### 4.6. Predicting Patient Response to Immune Checkpoint Inhibitor Therapy

To categorize samples into responders and non-responders, we used response evaluation criteria in solid tumors (RECIST), where complete responders (CR) and partial responders (PR) were categorized as responders, and stable disease (SD) and progressive disease (PD) were categorized as non-responders.

To evaluate the ability of the ICP-related lncRNAs to predict ICI response, five datasets of melanoma treated with immunotherapy were obtained (Supplementary Table S2). The batch effects were removed using the R package ‘sva’ (Version 3.46.0). Seventy percent of the samples were used as the training set, and the remaining samples were used as the test set. We further tested the predictive performance of the models using random forest (RF), extreme gradient boosting (XGBoost), and support vector machine (SVM) analysis. We compared the predictive performance of models based on ICP-related lncRNAs and other clinical indicators (PD-1, PD-L1, and CTLA4) using SVM models (Version 1.17.3). The ‘ROCR’ R package (Version 1.0.11) was used for receiver operating characteristic (ROC) curves to evaluate the predictive power of the model.

### 4.7. Statistical Analysis

Statistical analyses were conducted using R software version 4.2.2 and 4.3.1. The statistical significance of differences between groups was evaluated using the Wilcoxon Mann–Whitney test. The Kruskal–Wallis test was used to compare the statistical significance of samples between multiple groups. Chi-squared test and Fisher’s exact test were used to determine whether or not there was a significant association between two categorical variables. The survival difference between groups was assessed by log-rank test. A *p*-value < 0.05 was considered statistically significant (* *p* < 0.05; ** *p* < 0.01; *** *p* < 0.001; **** *p* < 0.0001).

## 5. Conclusions

In summary, we revealed the associations between ICPs, lncRNAs, and immune in melanoma by integrating bulk, single-cell, and immune cell line datasets. ICP-related lncRNAs could better predict immunotherapy response than single-molecule therapy in melanoma patients. These findings may provide effective candidates for further exploration of the immune function and regulation of lncRNAs and will be valuable in future immunotherapy strategies.

## Data Availability

The data that support the findings of this study are available from The Cancer Genome Atlas (TCGA, https://portal.gdc.cancer.gov/, accessed on 1 November 2022). Healthy human skin tissue data from Genotype Tissue Expression (GTEx) were downloaded from the UCSC Xena platform (http://xena.ucsc.edu/, accessed on 11 November 2022). Immune cell expression profiles were obtained from Gene Expression Omnibus (GEO, https://www.ncbi.nlm.nih.gov/geo/, accessed on 10 November 2022) with the accession numbers GSE6863 [24], GSE8059 [25], GSE13906 [26], GSE23371 [27], GSE25320 [28], GSE27291 [29], GSE27838 [30], GSE28490 [31], GSE28698 [32], GSE28726 [33], GSE37750 [34], GSE39889 [35], GSE42058 [36], GSE49910 [37], GSE51540 [38], and GSE59237 [39]. GSE115978 [49] was used to perform scRNA-seq expression profiles. Immunotherapy response data [51,52,53] was obtained from Tumor-Immune Dysfunction and Exclusion (http://tide.dfci.harvard.edu/, accessed on 1 June 2023). Code Availability: The code used in the work is available on GitHub (https://github.com/luqianyiouo/sweetie, accessed on 15 April 2025). All software tools used in this study are freely available.

## Supplementary Materials

The following supporting information can be downloaded at: https://www.mdpi.com/article/10.3390/ijms26104557/s1.

## Abbreviations

AUC: area under the curve; lncRNAs: long non-coding RNAs; ICP: immune checkpoints; ICI: immune checkpoint inhibitors; TCGA: The Cancer Genome Atlas; GEO: Gene Expression Omnibus; GSEA: gene set enrichment analysis; GTEx: Genotype-Tissue Expression; OS: overall survival; PCC: partial correlation coefficient; RF: random forest; ROC: receiver operating characteristic; SKCM: skin cutaneous melanoma; SVM: support vector machine; tSNE: t-distributed Stochastic Neighbor Embedding; XsGBoost: extreme gradient boosting
