# Supplementary material for: NetLnc: A Network-Based Computational Framework to Identify Immune Checkpoint-Related lncRNAs for Immunotherapy Response in Melanoma
Source: Int J Mol Sci. 2025 May 9;26(10):4557. doi: 10.3390/ijms26104557 (PMC12110832; doi:10.3390/ijms26104557)
Supplement: Supplementary file 1 [file ijms-26-04557-s001.zip › ijms-3561391-supplementary.pdf]

Supplementary Table S1. Datasets of 19 immune cell lines

Cell Type	Dataset		Sample
B cell activated	GSE28490		5
CD4 T cell activated	GSE28726	GSE49910	7
CD4 T cell resting	GSE28726		4
CD8 T cell activated	GSE49910		6
CD8 T cell resting	GSE49910		4
Dendritic cells activated	GSE59237		4
Dendritic cells resting	GSE59237		6
Eosinophils	GSE28698		3
Immature dendritic cells	GSE6863	GSE23371	6
Mast cells activated	GSE25320		4
Monocytes	GSE49910		6
Myeloid dendritic cells	GSE42058		4
Neutrophils	GSE39889	GSE49910	7
NK activated	GSE8059	GSE27838	11
NK resting	GSE8059		1
NKT activated	GSE28726		6
Plasmacytoid dendritic cells	GSE37750		8
T gamma delta	GSE13906	GSE27291	10
T helper 17	GSE51540		9

Supplementary Table S2. Datasets used for ICI response analysis

Cohort	Treatment	Sample	Responder	Non-responder	Therapy
Gide et al.	anti-CTLA-4+PD-1	32	21	11	nivolumab or pembrolizumab combined with ipilimumab
Gide et al.	anti-PD-1	41	19	22	nivolumab or pembrolizumab
Van Allen et al.	anti-CTLA-4	37	14	23	ipilimumab
Riaz et al.	anti-PD-1	25	6	19	ipilimumab-naive
Riaz et al.	anti-PD-1	26	4	22	ipilimumab-prog

Supplementary Figure S1

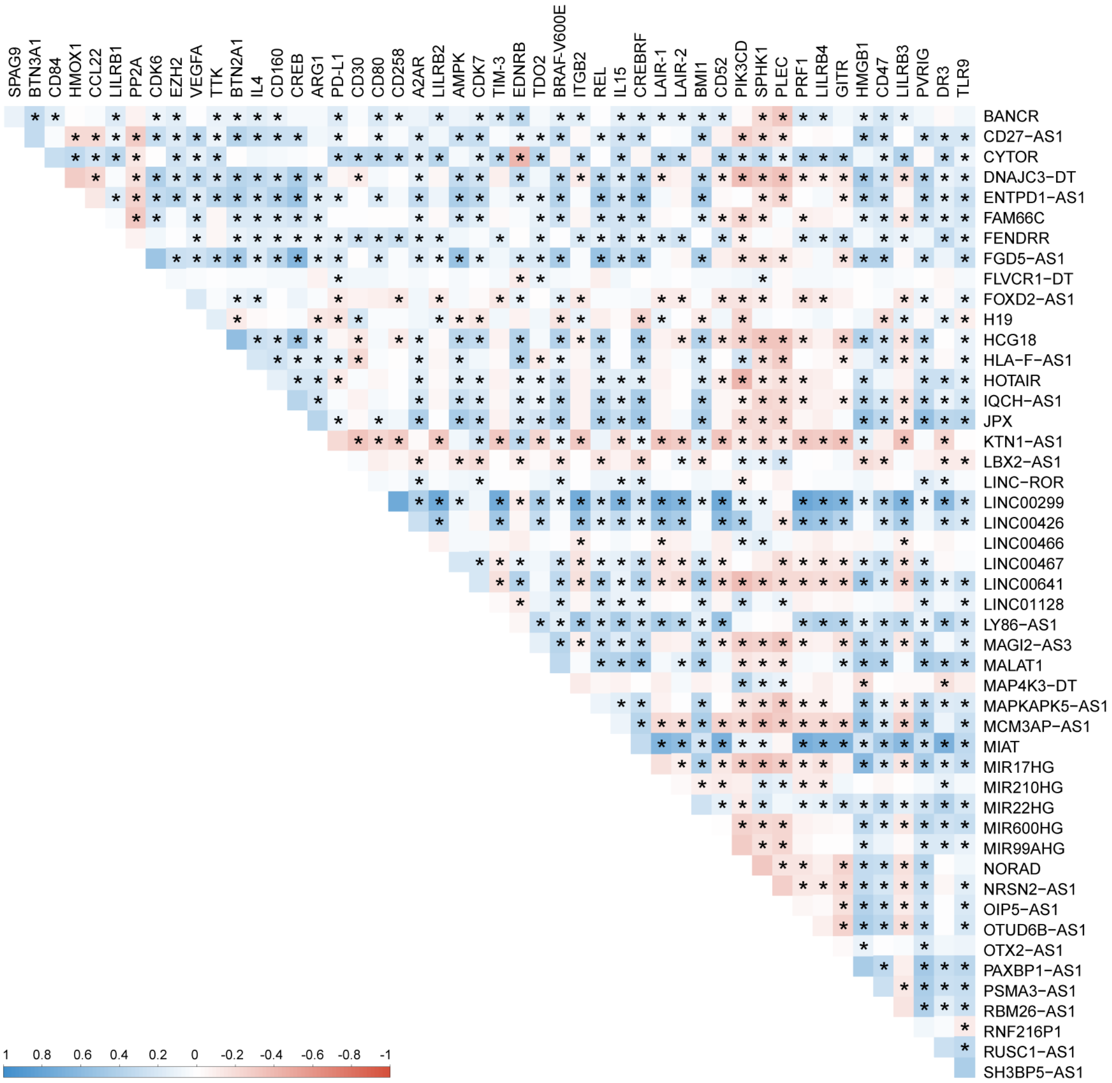

Supplementary Figure S1. The Spearman Correlation between lncRNAs and ICPs. (*$p < 0.05$)

Supplementary Figure S2

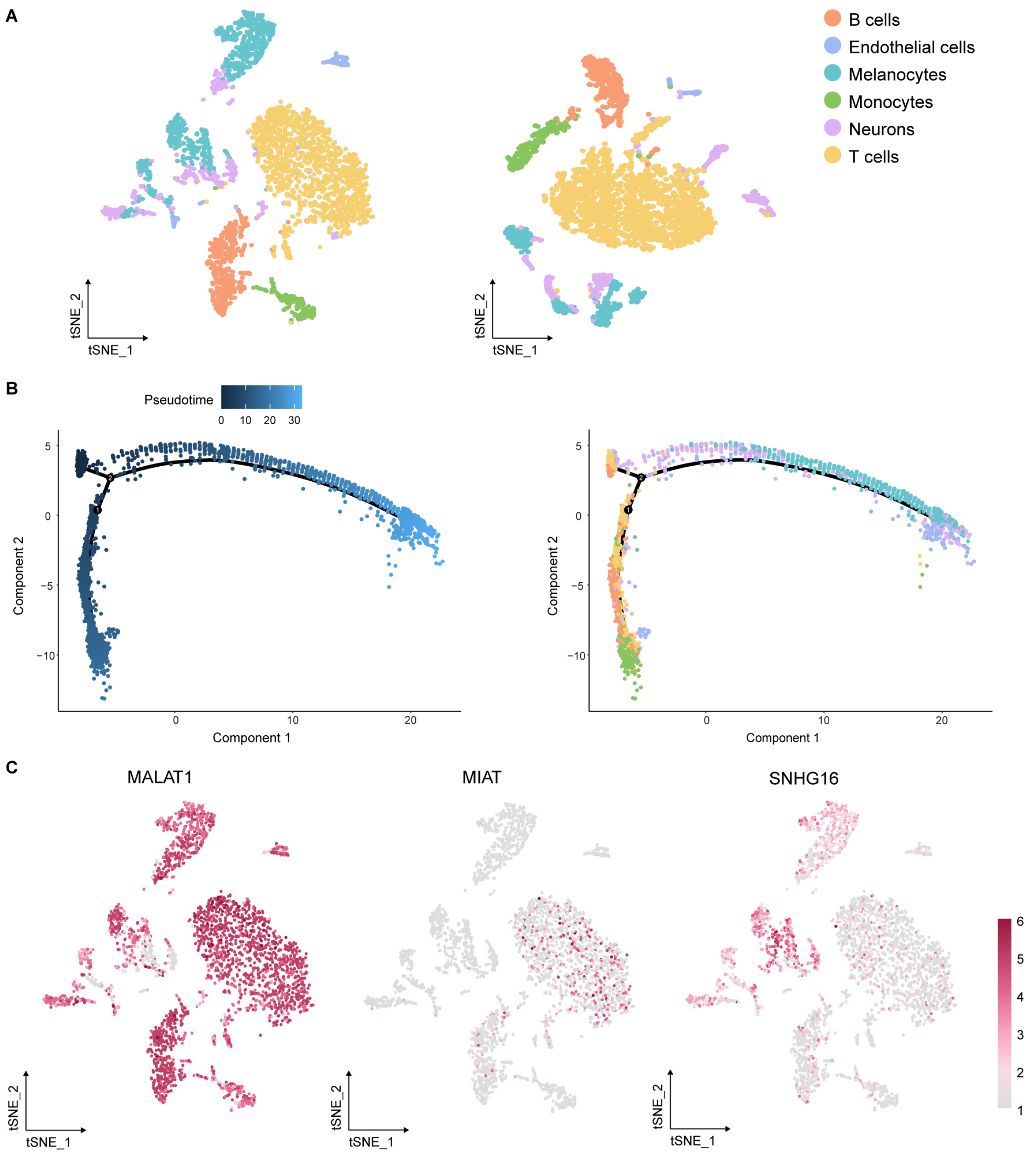

Supplementary Figure S2. (A) tSNE plots of cells in melanoma before and after immunotherapy. **(B)** Discriminative dimensionality reduction (DDR) tree visualization of trajectory with mapping of pseudotime. **(C)** tSNE plots of lncRNA expression in melanoma.

Supplementary Figure S3

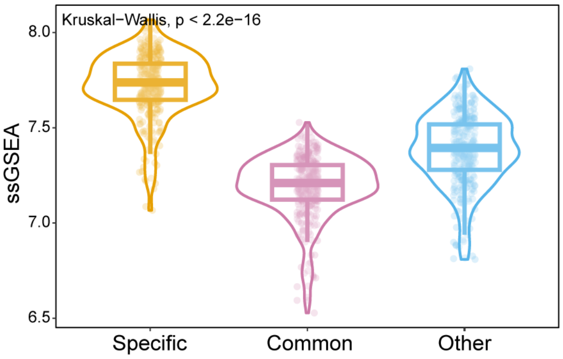

Supplementary Figure S3. Violin plots representing the overall comparison in ssGSEA scores for lncRNA-ICP pairs with different patterns. Boxes indicate the interquartile range of the data.

Supplementary Figure S4

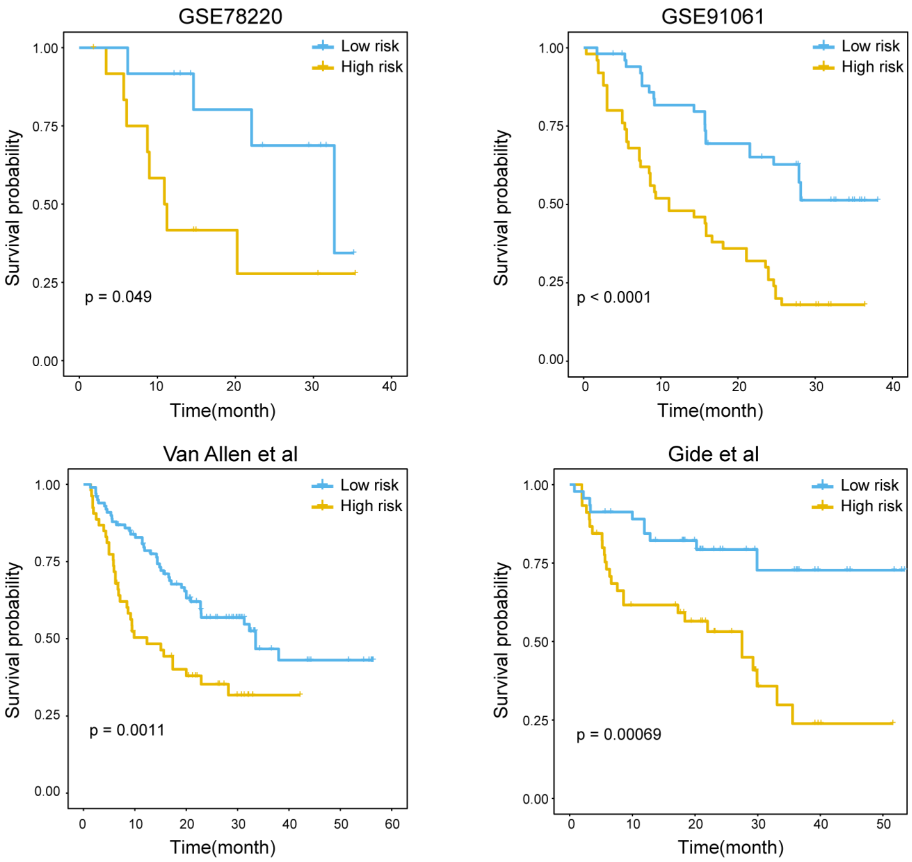

Supplementary Figure S4. Kaplan-Meier survival analysis of OS for patients with high (yellow) and low (blue) risk scores. The survival difference is calculated by log-rank test.
